# Physiological phenotyping of transpiration response to vapour pressure deficit in wheat

**DOI:** 10.1186/s12870-024-05692-3

**Published:** 2024-10-30

**Authors:** Anna Moritz, Andreas Eckert, Stjepan Vukasovic, Rod Snowdon, Andreas Stahl

**Affiliations:** 1 Department of Plant Breeding, Justus Liebig University Giessen, Giessen, Germany; 2 Institute for Resistance Research and Stress Tolerance, Julius Kühn Institute (JKI) – Federal Research Centre for Cultivated Plants, Quedlinburg, Germany

**Keywords:** Drought stress, High-throughput phenotyping, Water use efficiency, Transpiration restriction, Vapour pressure deficit, Wheat

## Abstract

**Background:**

Precision phenotyping of short-term transpiration response to environmental conditions and transpiration patterns throughout wheat development enables a better understanding of specific trait compositions that lead to improved transpiration efficiency. Transpiration and related traits were evaluated in a set of 79 winter wheat lines using the custom-built “DroughtSpotter XXL” facility. The 120 l plant growth containers implemented in this phenotyping platform enable gravimetric quantification of water use in real-time under semi-controlled, yet field-like conditions across the entire crop life cycle.

**Results:**

The resulting high-resolution data enabled identification of significant developmental stage-specific variation for genotype rankings in transpiration efficiency. In addition, for all examined genotypes we identified the genotype-specific breakpoint in transpiration in response to increasing vapour pressure deficit, with breakpoints ranging between 2.75 and 4.1 kPa.

**Conclusion:**

Continuous monitoring of transpiration efficiency and diurnal transpiration patterns enables identification of hidden, heritable genotypic variation for transpiration traits relevant for wheat under drought stress. Since the unique experimental setup mimics field-like growth conditions, the results of this study have good transferability to field conditions.

**Supplementary Information:**

The online version contains supplementary material available at 10.1186/s12870-024-05692-3.

## Background

Climate change is expected to cause increasing temperatures and altered precipitation patterns [1]. Although for many regions the absolute annual precipitation amounts may not necessarily diminish substantially, phases of heavier rainfall may alternate with periods of drought, resulting in an unfavourable distribution of water availability. This is critical for plant development as insufficient water availability at sensitive developmental stages can severely impact plant performance. Due to increasing temperatures and decreasing relative humidity, the vapour pressure deficit (VPD) is projected to increase in many regions. This will raise the evaporative demand, promoting consumption of soil water [2] and limiting its availability in subsequent phases.

The breeding of adapted varieties is seen as a key strategy to sustain crop production in water-limited scenarios. Classical selection, more recently supported by genomic prediction models, has been successful in increasing varietal performance even under water deficit [3]. But it is desirable that selection for specific traits surpasses a “black box” approach. However, the response to and productivity under insufficient water supply are genetically extremely complex, characterised by strong genotype-by-environment interactions and quantitative inheritance [4].

To describe water productivity several definitions exist. Commonly, the term “water use efficiency” (WUE) in agricultural systems is defined as the ratio between grain yield and the amount of water received during the development [5], i.e. the evapotranspiration, which is composed of the transpiration of the crop and water that is lost by evaporation. This definition shows that WUE can be improved through effective crop management practices [6] such as adjusting the sowing date, implementing weed control and optimizing plant density. This term “WUE” is often used to describe water productivity at the canopy level. Transpiration efficiency (TE), on the other hand, is usually defined as the ratio between the biomass of the crop and the water transpired by the crop. The “intrinsic WUE” has been described as the ratio of CO_2_ assimilation and transpiration at the leaf level [5]. Yield performance under water-limited conditions is often described by the “Passioura equation”, in which grain yield is expressed as the product of water uptake (WU), TE and harvest index (HI) [7]. These three factors still represent complex aggregated traits that are expressed at different developmental stages and are not completely independent of each other. Considering the response to drought stress is not static, but rather highly dynamic, the approach is not granular enough to understand the response of different genotypes during specific periods of stress and to identify the responsible subtraits. Ideally, useful subtraits should be determined by a smaller number of genes and show a higher heritability than highly quantitative traits (such as yield or TE), enabling their use to positively affect selection accuracy and thus contribute to accelerated breeding progress. This implies a need to identify such subtraits that show a higher heritability and strong correlation to TE and WU, but are also relatively simple to measure precisely.

Various organs regulate the plant’s water balance. Several characteristics of the root system determine WU [8]. For example, morphological root traits, such as root depth and density, impact WU from different (especially deeper) soil layers [9, 10]. Physiological root traits controlling root conductance, including aquaporins [11–13] or apoplastic barriers [14] as well as the diameter of the metaxylem, are associated with crop water status and TE [11, 15]. The release of water is largely determined by the leaf surface and the density of the stomata. Root system and leaf surface are not dynamically adaptable at the onset of stress and therefore have to be considered as constitutive and usually irreversible factors of water balance regulation, which can only be adjusted in the long term. In contrast, stomata, as the sites of gas exchange, are particularly important for short-term regulation of transpiration.

A fundamental conflict for crop productivity under water-limited conditions is the common pathway of water and carbon exchange between the leaf and the atmosphere: When stomata are opened for carbon fixation, water is transpired as well. VPD plays a major role in this context. In addition to stomatal conductance, VPD acts as a driving force on transpiration. An increase in VPD leads to a higher water consumption per unit of fixed CO_2_. To prevent excessive water loss and to counteract dehydration and senescence, the plant is able to close its stomata, which in turn causes a decrease in photosynthetic rate. This behaviour, i.e., restricting transpiration when VPD is adverse, enables the plant to conserve soil water [16, 17], thereby improving TE [18]. Water conserved in this manner is available for the plant at later time points, enabling maintenance of physiological activity for an extended period of time [2, 19, 20]. This trait, e.g. the limitation of transpiration rate in response to increasing VPD has been observed in several crop species [21]. The physiological basis of transpiration restriction in response to high VPD is assumed to be related to a reduced hydraulic conductivity which is associated with aquaporin activity [21]. Studies on several crop species linked (e.g. 21–25) the limitation of transpiration to aquaporin expression. Simulations have demonstrated that the ability of wheat plants to restrict their transpiration at higher VPD levels contributes to a yield gain of 2.5% on a national average in Australia. The strongest yield gains were observed in the northeast of the country, where crops are dependent on soil water during the grain filling phase [26]. It has been shown that wheat breeding in Australia over the past 120 years has indirectly selected cultivars with an improved ability to restrict transpiration under unfavourable VPD conditions [27]. Restricted transpiration response to increasing VPD has been repeatedly associated with increased crop production under water-limited environments [5, 28–30]. Nevertheless, the effect of this trait on crop productivity depends on the environmental conditions in which the crops are cultivated. Under water-limited conditions, plants showing this trait might have an advantage, but under optimal conditions genotypes that do not restrict their transpiration might perform better [31]. Hence, short-term adaptation to variations in VPD is of particular interest, as it enables a flexible, short-term adaptation to unfavourable conditions.

In the past years, the innovative concept of “functional physiological phenotyping” [32] has introduced advanced opportunities for phenotyping of the dynamic physiological responses of the crop to fluctuating environmental conditions. The basis of this concept is the continuous phenotyping of physiological traits in a non-destructive and high-throughput manner, while simultaneously collecting information on environmental conditions to which the crop is exposed. This resulting comprehensive data is the basis for statistical analysis to draw conclusions to compare the genotype specific responses to changes in the environmental conditions in order to identify superior genotypes or understand the underlying mechanisms that lead to an improved performance, especially under drought stress conditions [32].

In light of strong genotype-by-environment interactions, a suitable phenotyping environment is required for such physiological phenotyping approaches to detect and understand subtle differences in transpiration behaviour [32–34]. Since the field is the target environment, field trials are irreplaceable to obtain relevant information on crop performance under realistic scenarios, especially considering complex genotype-by-environment interactions [35]. Traits like transpiration are extremely difficult to capture precisely in the field. In particular, precise, direct quantification of transpiration dynamics in “real-time” is barely feasible in the field. In addition, onset and intensity of a drought period cannot be controlled in a normal field environment. However, targeting drought stress tolerance traits requires clearly-defined drought stress conditions “on demand” [36]. Controlled environments meet these requirements, however the conditions in greenhouses and growth chambers are normally too artificial to enable a successful extrapolation of findings into field-relevant conclusions. There are numerous reasons for this, particularly inadequate pot size, lighting, crop density and temperature control [37, 38]. To overcome those limitations, the use of large containers, which enable field-like crop densities and sufficient root depth, has been suggested as a compromise between field and controlled environments [39]. Complemented by digital high-throughput methods to assess plant growth non-destructively across the entire crop development [40, 41], along with gravimetric monitoring of pot weights [33, 39, 42, 43], such systems allow reliable and precise assessment of WU and TE and constant monitoring of diurnal transpiration patterns in combination with a high significance for field environments.

In this study we collected high-resolution data describing genetic variation for real-time transpiration efficiency and the dynamic transpiration response to ambient vapour pressure deficit in a broad collection of 79 winter wheat genotypes in the DroughtSpotter XXL facility, which aims at simulating field conditions. This enabled a highly detailed investigation of the genotypic response to elevated VPD under realistic growth conditions. A large panel of wheat genotypes adapted to central European climate conditions was investigated, including numerous recent, high-performing elite breeding lines. Results provide deep insight into the mechanism contributing to one aspect of transpiration efficiency and have key significance for future breeding in the face of climate change.

## Materials and methods

### Genotypic material

The study used a panel of 79 winter wheat breeding lines (*Triticum aestivum* L.) originating from crosses between modern elite winter wheat varieties adapted to Western European growing conditions. All lines were derived from advanced phases of commercial winter wheat breeding programs. The population structure of the tested genotypes in form of a principal component analysis based on SNP markers is illustrated in Supplementary Fig. S1.

### Trial under semi-controlled environment

#### DroughtSpotter XXL facility

**Fig. 1:**
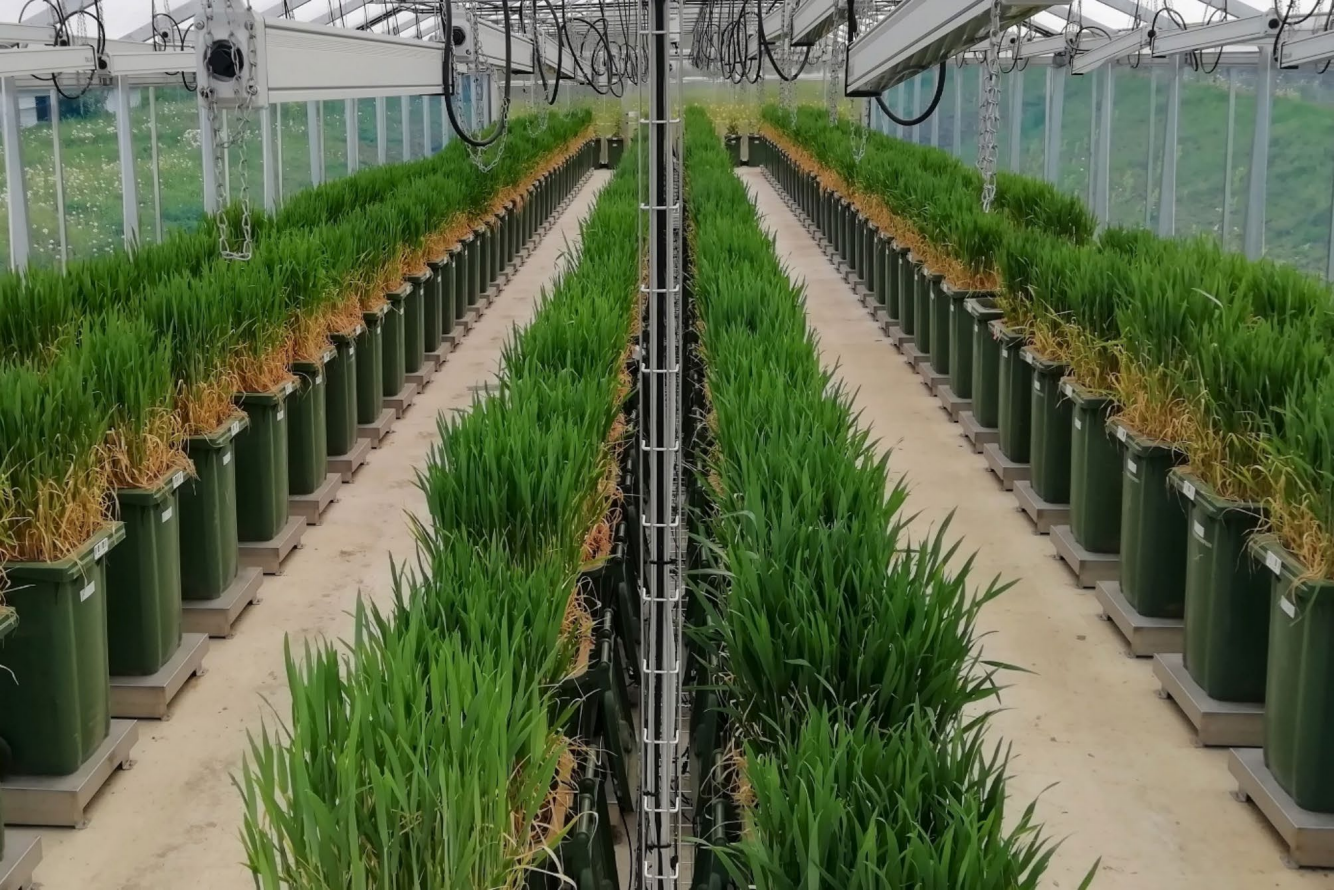
DroughtSpotter XXL precision phenotyping platform. The facility comprises 240 high-resolution gravimetric scales on which containers with a soil volume of 120 l and a depth of 90 cm are placed. Transpiration is monitored gravimetrically in 5-minute intervals over the entire lifecycle of the crop. The facility is situated inside of a greenhouse with open walls

Experiments were conducted in the plant phenotyping platform DroughtSpotter XXL (Phenospex, Heerlen Netherlands) at the Rauischholzhausen field station of Justus Liebig University in Giessen, Germany (N 50° 45′ 39″ E 8° 52′ 47″, 235 m a.s.l). The DroughtSpotter XXL comprises 240 large plant containers with a volume of 120 l, a height of 90 cm and feature a planting area of 0.16 m^2^ (also see [36, 44]). Each container was filled with 155.5 kg sand-soil mixture at ratio 3:2 and placed on a high-resolution gravimetric scale which recorded the weight of each individual container every five minutes throughout the entire vegetation period (Supplementary Fig. S2). To ensure measurement accuracy and minimize noise, the power supply of the junction boxes was separated from the control unit in order to avoid interference due to changing voltage. This set-up facilitated continuous, high-resolution monitoring of plant transpiration for every individual genotype. A total of three evaporation checks, containing no plants were included. The evaporation checks were treated identically to the experimental containers, enabling quantification of evaporation from the soil surface so that transpiration and evaporation could be measured separately. The facility was located in a plastic greenhouse (360 m^2^) (Fig. 1) to protect the containers from rain. The sides of the greenhouse were opened to expose the plants to natural temperature and wind fluctuations as in the field (the ceiling remained closed at all times).

#### Plant cultivation

Automated irrigation took place daily after midnight (time of minimum transpiration). Each container was individually irrigated to a target weight corresponding to a predefined target field water capacity of 60%. The weight of the container that corresponds to 100% field capacity is defined as the weight of the water that the soil could hold after two days against gravity. This was determined by using two soil-filled containers that were perforated at the bottom before the main experiment and placed on gravimetric scales. These containers were very slowly and carefully saturated with water until water leakage was observed, at which point the weight at 100% field capacity was recorded and the target weight for other field capacities was calculated proportionally. Recognizing that completely saturating the soil with water might require multiple irrigations, water was added carefully over a period. Given that drier soil tends to seep very quickly, the initial irrigation was executed deliberately, to ensure a gradual increase in water content until saturation is obtained. Additionally, the soil of all containers of the main experiment was carefully mixed with water before planting over an elongated period to guarantee the correct water content in the soil.

Sowing was conducted on 14 December 2020 with 63 seeds per container distributed equally in three rows. After vernalization (79 days after sowing), the number of plants per container was reduced to 48 plants per container (3 rows with 16 plants each). The experiment was designed as an alpha-lattice with three replications per genotype. Fertilization was conducted according to Supplementary Table S1 and plant protection was applied according to the occurrence of disease pressure in the greenhouse in order to avoid biotic stress.

**Fig. 2:**
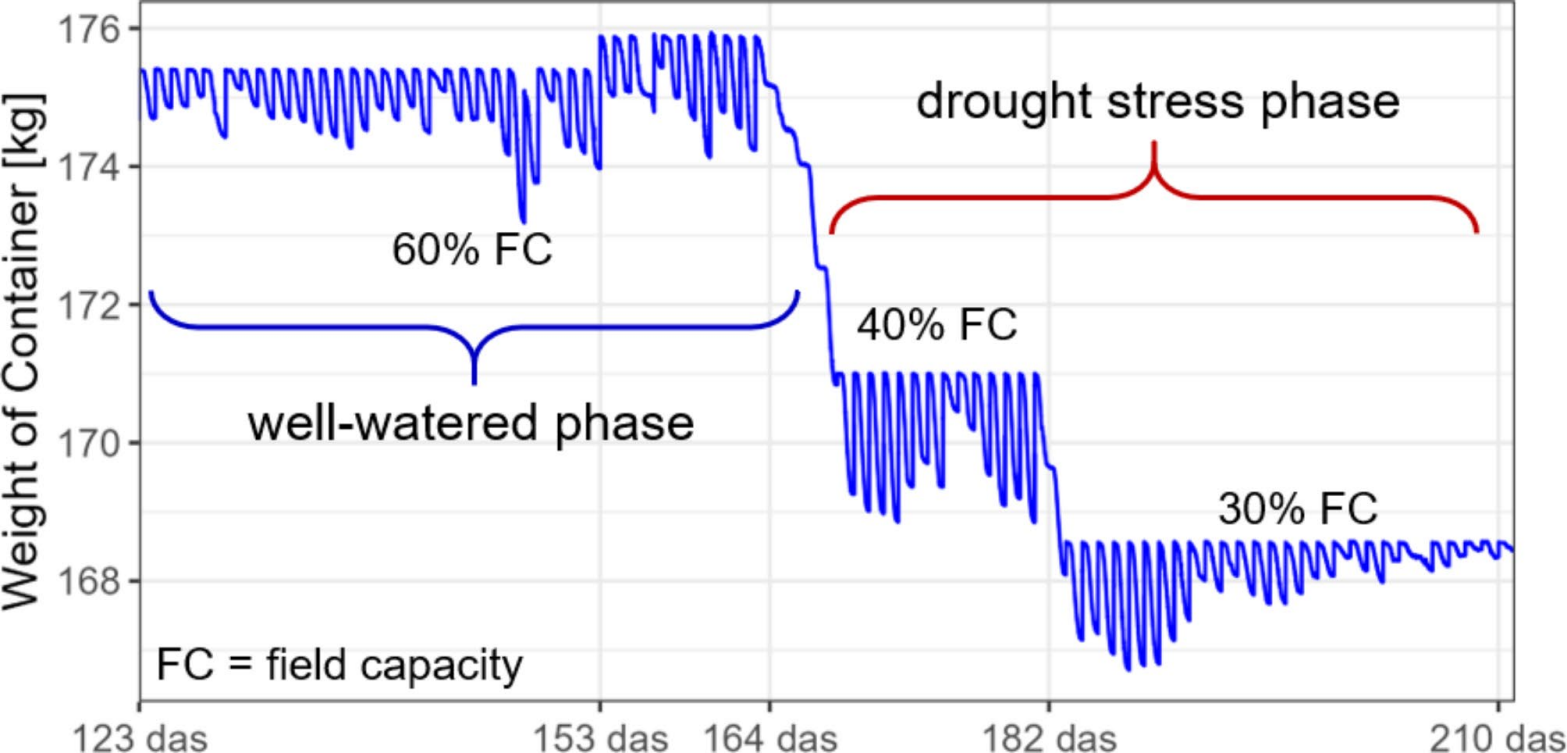
Irrigation scheme for the container trial. The container weight of a single container is displayed for the period between 123 days after sowing (das) and 210 das. Every day after midnight, the container was irrigated up to a predefined target weight, resulting in a rapid increase in container weight. Over the course of the day the plants transpired water, resulting in a gradual decrease of the container weight. At 164 das drought stress was applied by reducing field capacity to 40% and at 182 das drought stress conditions were intensified by setting field capacity to 30%

At the beginning of the experiment, all containers were irrigated to 60% field capacity for a well-watered phase. At 153 days after sowing (das), the target container weight of all containers was increased by 480 g to account for plant biomass growth. The adjustment corresponds to the average weight of the plant harvested from three containers separate from the main experiment, with the lines grown in these containers selected from the full panel of genotypes. At the beginning of flowering (164 days after sowing) a drought stress phase was initiated by reducing irrigation to 40% field capacity. 18 days after initial drought stress application (182 days after sowing), drought stress conditions were intensified by reducing the field capacity to 30% (Fig. 2). Irrigation to 30% field capacity was then continued until harvest.

#### Determination of plant biomass, grain yield and primary yield components

Digital biomass, leaf area, normalized vegetation difference index (NDVI) and green leaf index (GLI) were assessed weekly from tillering to harvest using a three-dimensional dual-laser scanner (Plant Eye F500, Phenospex). For this purpose, the scanning trolley was placed in front of each container and the two cameras, mounted parallelly on a rail, scanned the plant vertically from the upper edge of the container (Supplementary Fig. S3). The two cameras have a slight angle to each other so that one camera can capture pixels that are in the shadow of other plants and are not detected by the other camera. By sensor fusion, images were automatically merged into a 3D image [40, 45]. Based on preliminary tests, in which the digital values were checked by manual biomass and height determinations (Supplementary Fig. S4 and Supplementary Fig. S5), a good reliability of the digital acquisition method could be confirmed (R² = 0.89 for plant height and R² = 0.51 for plant biomass).

Harvest took place on 22 July 2021 (220 days). The total above-ground biomass per container was harvested. The plants were threshed and grain yield was determined along with the number of spikes per container, thousand grain weight (TGW), grains per spike and total biomass yield per container.

#### Assessment of environmental metadata

Air temperature and relative humidity within the DroughtSpotter XXL facility was assessed using data loggers (PCE-HT71N, PCE Instruments). These data loggers offer an accuracy of ± 1 °C for temperature and ± 3% for relative humidity and a resolution of 0.1 °C and 0.1% for temperature and relative humidity, respectively. To assess temperature and humidity gradients within the greenhouse, nine of the above mentioned data loggers were placed symmetrically across all rows and columns in a 3 × 3 grid. The data loggers recorded air temperature and relative humidity every ten minutes.

### Data analysis

#### Data pre-processing

Daily evapotranspiration per container was calculated based on the weight recordings of the DroughtSpotter XXL, using the difference of the container weight after irrigation and before the subsequent irrigation. Daily evaporation of the evapo-check containers was determined the same way. Subsequently, the amount of transpiration per container was determined by subtracting the values for the daily evapotranspiration for the mean daily amount of evaporation. From these daily transpiration amounts cumulative transpiration over the complete trial period (or other specific periods of interest) was calculated. For the transpiration rate (TR) in g per container per minute, weight curves were smoothed using a Loess-function and the values were interpolated every minute. TR was normalized for leaf area.

#### Calculation of transpiration efficiency

To estimate TE at any given timepoint during crop development, digital biomass data from the weekly scans was interpolated to obtain daily values and relate these with the daily recordings for transpiration. The developmental stage-specific transpiration efficiency ($\:{d}{{TE}}_{{t}}$) is defined as the digital biomass estimated by the 3D laser scanner up to a certain point in time per unit of transpired water up to the same point in time, calculated with Eq. 1.

1 $$\:{d}{{TE}}_{{t}}{=}\frac{{{dBM}}_{{t}}{\:}\left[{{dm}}^{{3}}\right]}{{{T}}_{{t}}{\:}{[}{{dm}}^{{3}}{]}}$$

where $\:{{dBM}}_{{t}}$ is the digital biomass at a given point in time and $\:{{T}}_{{t}}$ is the amount of water transpired up to this point in time.

The overall *TE* is defined in Eq. 2 as ratio of grain yield ($\:{GY}$) and the total amount of transpired water ($\:{T}$):

2 $$\:{TE=}\frac{{GY\:[g]}}{{T\:[kg]}}$$

#### Response of transpiration rate to VPD

The recordings for temperature ($\:{Temp}$ in °C) and relative humidity ($\:{RH}$ in %) of the nine data loggers were used in Eq. 3 to calculate VPD:

3 $$\:{VPD=}\frac{{100-RH}}{{100}}{\times0.6108{\times}}\,{{e}}^{{17.2*Temp(Temp+237.3)}}$$

Based on the values for VPD from the nine datalogger positions, VPD values for the position of each container were interpolated.

Response of TR to VPD is commonly examined via a segmented linear regression [27, 31, 46] which determines the breakpoint (BP) VPD value above which transpiration is restricted. Additionally, two slopes are determined: Slope 1, characterizing the response of TR to VPD values below the breakpoint, and Slope 2, characterizing the response of TR to VPD values above the breakpoint. ΔSlope, calculated as the difference between Slope 1 and Slope 2, characterizes the strength of transpiration restriction. Since the transpiration response to VPD was measured under ambient uncontrolled conditions, only days where VPD exceeded 3 kPa were considered for evaluation, as on cool and moist days with low VPD, no restriction of transpiration in response to VPD is expected. This was the case for six days during grain filling (180 das, 184 das, 185 das, 186 das, 187 das and 189 das). Furthermore, only the timeframe between 6:00 and 17:00 was considered. The segmented regression analysis was performed for each container individually. Starting from a linear model (Y = Slope × X + Intercept) we first tested whether a significant change in slope could be observed for the relationship between VPD and TR. In cases where the majority of container-day-combinations for one genotype showed a significant change in slope, the genotype was considered to have a segmented transpiration response to VPD. If a small fraction of observations for one genotype did not show a significant breakpoint, the corresponding observations were excluded from further evaluation. All genotypes expressed a predominant pattern of a segmented transpiration response. The segmented linear regression model (for X < BP: Y1 = Slope1 × X + Intercept1 and for X > BP: Y2 = Slope2 × X + Intercept2) was then applied to estimate BP, Slope 1, Slope 2, and ΔSlope. If one of the parameters resulting from the segmented regression was lower than Q1–1.5 IQR or higher than Q3 + 1.5 IQR, it was declared as an outlier and the complete segmented regression analysis for this observation was excluded from further analysis. Additionally, segmented regression models showing a low fit (R^2^ < 0.5; 16 observations) were excluded.

#### Linear mixed model analysis and repeatability estimation

Linear mixed models allow for both fixed and random effects. Here, linear mixed models were applied to estimate adjusted means for BP, Slope 1, Slope 2, and ΔSlope across three replications and six days for each genotype. The following linear mixed model was applied (Eq. 4):

4 $$\:{{P}}_{{ijklmn}}{=\mu+}{{g}}_{{i}}{+}{{D}}_{{j}}{+}{\left({GD}\right)}_{{ij}}{+}{{B}}_{{k}}{+}{{Z}}_{{l}}{+}{{C}}_{{m}}{+}{{R}}_{{n}}{+}{{e}}_{{ijklmn}}$$

where $\:{{P}}_{{ijklmn}}$ represents the observed phenotype, $\:{\mu}$ is the general mean, $\:{{g}}_{{i}}$ is the fixed effect of the $\:{i}$th genotype, $\:{{D}}_{{j}}$ is the random effect of the $\:{j}$th day from which the observation originates, $\:{{(GD)}}_{{ij}}$ is the random effect of genotype $\:\:{i}$ within day $\:{j}$, $\:{{B}}_{{k}}$ is the random effect of the $\:{k}$th block, $\:{{Z}}_{{l}}$ is the random effect for the $\:{\:l}$th row, $\:{{C}}_{{m}}$ is the random effect of the $\:{m}$th column, $\:{{R}}_{{n}}$ is the random effect of the $\:n$th replication and $\:{{e}}_{{ijklmn}}$ is the error term.

In order to estimate repeatability, the genetic variance and the standard error of the difference between the means is required [47]. To estimate variance components, including the genetic variance, for the transpiration response traits, a modified version of the model in Eq. 4 was used with all factors random (Eq. 5):

5 $$\:{{P}}_{{ijklmn}}{=\mu+}{{G}}_{{i}}{+}{{D}}_{{j}}{+}{\left({GD}\right)}_{{ij}}{+}{{B}}_{{k}}{+}{{Z}}_{{l}}{+}{{C}}_{{m}}{+}{{R}}_{{n}}{+}{{e}}_{{ijklmn}}$$

where $\:{{P}}_{{ijklmn}}$ represents the observed phenotype, $\:{\mu}$ is the general mean, $\:{{G}}_{{i}}{\:}$is the random effect of the $\:{i}$th genotype, $\:{{D}}_{{j}}$ is the random effect of the $\:{j}$th day from which the observation originates, $\:{{(GD)}}_{{ij}}$ is the random effect of genotype $\:{i}$ within day $\:{j}$, $\:{{B}}_{{k}}$ is the random effect of the $\:{k}$th block, $\:{{Z}}_{{l}}$ is the random effect for the $\:{\:l}$th row, $\:{{C}}_{{m}}$ is the random effect of the $\:{m}$th column, $\:{{R}}_{{n}}$ is the random effect of the $\:{n}$th replication and $\:{{e}}_{{ijklmn}}$ is the error term.

The model in Eq. 6 was used to estimate the standard error of the difference between the means for repeatability ($\:{h}^{2}$) estimation.

6 $$\:{{P}}_{{ijklmn}}{=\mu+}{{g}}_{{i}}{+}{{d}}_{{j}}{+}{{b}}_{{k}}{+}{{z}}_{{l}}{+}{{c}}_{{m}}{+}{{r}}_{{n}}{+}{\left({GD}\right)}_{{ij}}{+}{{e}}_{{ijklmn}}$$

where $\:{{P}}_{{ijklmn}}$ represents the observed phenotype, $\:{\mu}$ is the general mean, $\:{{g}}_{{i}}{\:}$is the fixed effect of the $\:{i}$th genotype, $\:{{d}}_{{j}}$ is the fixed effect of the $\:{j}$th day from which the observation originates, $\:{{b}}_{{k}}$ is the fixed effect of the $\:{k}$th block, $\:{{z}}_{{l}}$ is the fixed effect for the$\:{\:l}$th row, $\:{{c}}_{{m}}$ is the fixed effect of the $\:{m}$th column, $\:{{r}}_{{n}}$ is the fixed effect of the $\:{n}$th replication, $\:{{(GD)}}_{{ij}}$ is the random effect of genotype $\:{i}$ within day $\:{j}$, and $\:{{e}}_{{ijklmn}}$ is the error term.

Subsequently, repeatability was estimated using Eq. 7:

7 $$\:{{h}}^{{2}}{=}\frac{{\sigma}_{{G}}^{{2}}}{{\sigma}_{{G}}^{{2}}{+}{{SE}}^{{2}}}$$

where *σ*^*2*^_*G*_ is the the genetic variance from Eq. 5 and SE is the standard error of the difference between the means from Eq. 6.

To estimate adjusted means for the traits that were assessed only once during the vegetation period, a different model was used. For all harvest traits and the TE from Eq. 2, the following model in Eq. 8 was used to estimate adjusted treatment means for each genotype across the three replicates:

8 $$\:{{P}}_{{iklmn}}{=\mu+}{{g}}_{{i}}{+}{{B}}_{{k}}{+}{{Z}}_{{l}}{+}{{C}}_{{m}}{+}{{R}}_{{n}}{+}{{e}}_{{iklmn}}$$

where $\:{{P}}_{{iklmn}}$ represents the observed phenotype, $\:{\mu}$ is the general mean, $\:{{g}}_{{i}}$ is the fixed effect of the $\:{i}$th genotype, $\:{{B}}_{{k}}$ is the random effect of the $\:{k}$th block, $\:{{Z}}_{{l}}$ is the random effect for the $\:{\:l}$th row, $\:{{C}}_{{m}}$ is the random effect of the $\:{m}$th column, $\:{{R}}_{{n}}$ is the random effect of the $\:{n}$th replication and $\:{{e}}_{{iklmn}}$ is the error term.

The model in Eq. 8, however with all factors random, was used to estimate genetic variance $\:{{\sigma}}_{{G}}^{{2}}$ and the error variance $\:{{\sigma}}_{{e}}^{{2}}$ to calculate repeatability for the endpoint traits in Eq. 9, where $\:{nrep}$ is the number of replications:

9 $$\:{{h}}^{{2}}{=}\frac{{\sigma}_{{G}}^{{2}}}{{\sigma}_{{G}}^{{2}}{+}{\sigma}_{{e}}^{{2}}\times\frac{{1}}{{nrep}}}$$

#### Clustering of genotypes with similar transpiration response to VPD

K-Means clustering was applied to classify genotypes based on their transpiration responses to VPD, in order to identify groups with distinct transpiration responses. The K-Means clustering was based on the value for breakpoint and ΔSlope, which represent key features from the segmented regression analysis. The optimal number of clusters was determined by assessing the within-cluster sum of squares.

#### Software

Data analysis was performed using the statistical programming language and environment R (version 4.1.1; R Core Team, 2021). The segmented regression analysis was performed using the R package *segmented* [48]. For modelling of the linear mixed effect models, the R package *lmerTest* [49] was used, and *emmeans* [50] was used to calculate adjusted means. The package *multcomp* [51] was used to create compact letter displays. Least significant differences (LSD) were calculated by multiplying the standard error of the difference of means with the 1 – α quantile of the normal distribution where α is 0.1.

## Results

### Genotypic variation in cumulative transpiration and transpiration efficiency

Among the 79 genotypes tested in the DroughtSpotter XXL trial, analysis of variance (ANOVA) revealed highly significant genotype effects on cumulative transpiration (*p* = 0.002) and TE (*p* = < 0.001), ranging between 62.11 and 89.86 kg and 2.12–3.00 g kg^− 1^, respectively (Table 1). Significant genotypic variation was also observed for total biomass (*p* = 0.049), grain yield (*p* = 0.001), yield components and HI (*p* = < 0.001) (Table 1). Repeatability for cumulative transpiration and TE was h^2^ = 0.45 and h^2^ = 0.71, respectively. As expected, transpiration was strongly correlated to grain yield (*r* = 0.64, *p* = < 0.001).

**Table 1:** Variation for endpoint traits from the wheat panel in the container trial

Trait	Min	Max	Mean	CoV	h^2^	Genotype
Cumulative transpiration [kg Container^− 1^]	62.11	89.86	74.83	0.08	0.45	**
Transpiration efficiency [g kg^− 1^]	2.12	3.00	2.54	0.06	0.71	***
Grain yield [g container^− 1^]	147.1	233.92	190.03	0.1	0.58	**
Total biomass [g container^− 1^]	397.96	542.22	473.52	0.07	0.37	*
TGW [g]	34.57	46.82	40.61	0.07	0.58	***
Spikes per container	84.98	143.13	117.55	0.11	0.77	***
Grains per spike	28.98	48.97	39.88	0.11	0.84	***
Harvest index	0.34	0.46	0.40	0.06	0.74	***

Minimum (Min), maximum (Max), mean and the coefficient of variation (CoV) of adjusted mean values for cumulative transpiration, transpiration efficiency, grain yield, total above-ground biomass, thousand grain weight (TGW), number of spikes per container, number of grains per spike and harvest index. Additionally, repeatability (h^2^) and the statistical significance of the genotypic effect are shown. *, *p* < 0.05; **, *p* < 0.01; ***, *p* < 0.001

### Developmental stage-specific response to drought stress

**Fig. 3:**
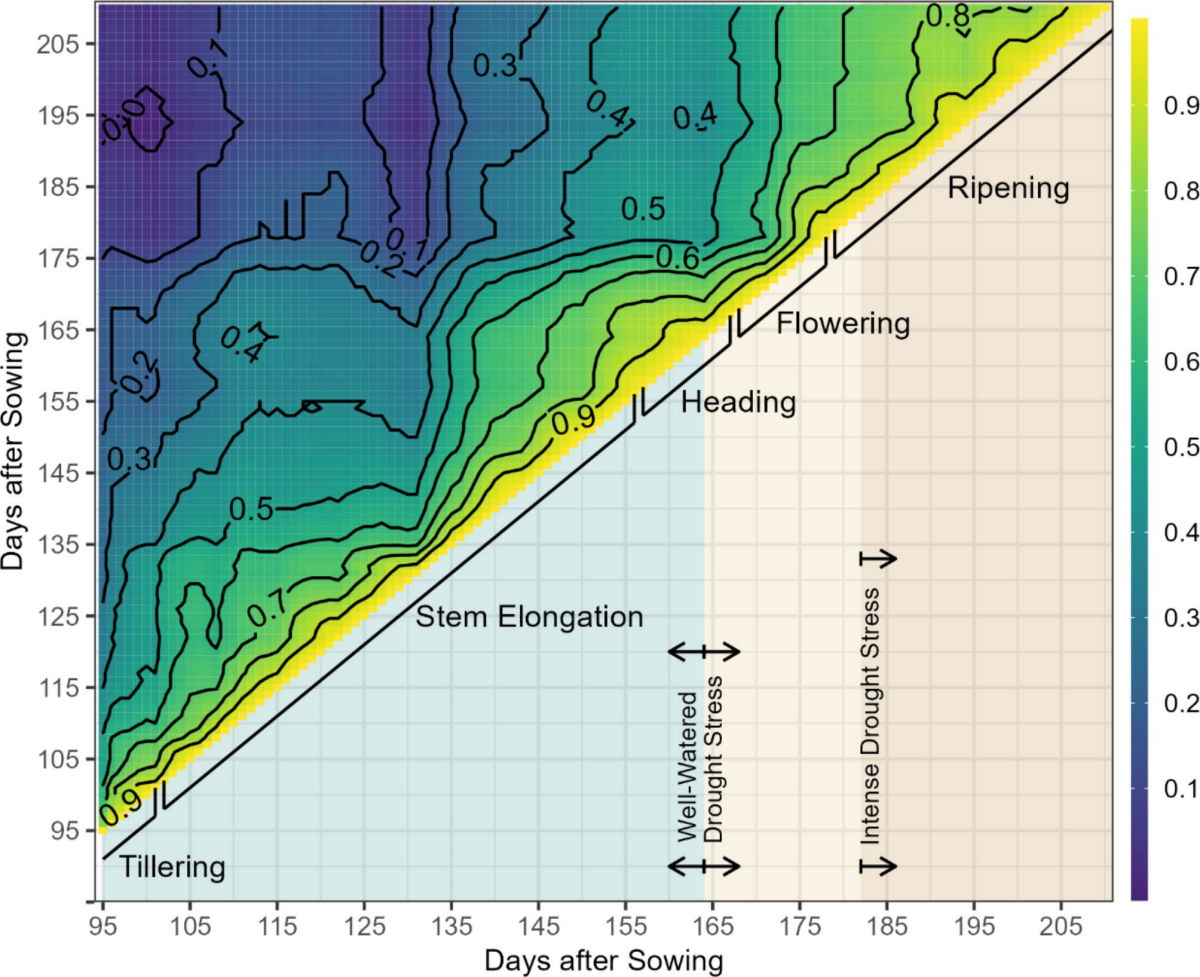
Kendall rank correlation for developmental-stage specific transpiration efficiency. The upper triangle shows the Kendall rank correlation coefficients for adjusted mean values between developmental-stage specific transpiration efficiency from 95 up to 211 days after sowing, with blue colours for weak correlations and yellow colours for strong correlations. The lower triangle provides information on developmental stages and drought stress conditions

Developmental-stage specific transpiration efficiency (dTE) is defined here as the ratio between the digital biomass estimated by the 3D scanner per unit of transpired water within a certain developmental stage. Analyzing Kendall rank correlation coefficients for dTE from 95 days after sowing until harvest revealed a consistently high correlation between dTE values over two consecutive days, but correlation between dTE values from earlier and later developmental stages was much weaker, indicating developmental stage-specific performance of the genotypes for dTE (Fig. 3).

Four macro stages were more closely evaluated: tillering, stem elongation, heading, flowering and ripening. ANOVA showed a highly significant genotype effect on dTE at each developmental stage (*p* = < 0.001) as well as a significant effect of genotype-by-developmental stage interaction (*p* = < 0.001), indicating developmental stage-specific variation for genotype rankings in TE. Repeatability for dTE at each developmental stage was high (h^2^ > 0.87) (Table 2).

**Table 2:** Variation for developmental-stage specific transpiration efficiency

Developmental Stage	Min	Max	Mean	CoV	h^2^
Tillering	17.6	39.5	26.8	0.19	0.88
Stem elongation	9.3	17.6	12.0	0.12	0.91
Heading	9.6	18.9	12.2	0.12	0.89
Flowering	8.1	17.0	11.7	0.13	0.89
Ripening	4.6	10.4	7.0	0.15	0.87

Minimum (Min), maximum (Max), mean and the coefficient of variation (CoV) of adjusted mean values for developmental-stage specific transpiration efficiency at the macro stages tillering, stem elongation heading flowering and ripening, calculated as in Eq. 1. Additionally, repeatability (h^2^) at each tested macro growth stage is shown

### Transpiration response to elevated VPD

During the six-day period (between 180 and 189 das) in which transpiration in response to VPD was evaluated, the temperature in the greenhouse ranged on average between 19.3 °C and 40.5 °C across all nine positions during daytime (06:00–17:00), while relative humidity ranged between 26.3% and 75.9%. This led to an average range in VPD across all nine positions from 0.55 kPa to 5.76 kPa (see Supplementary Fig. S2 for the range of temperature and VPD over the course of six days).

**Fig. 4:**
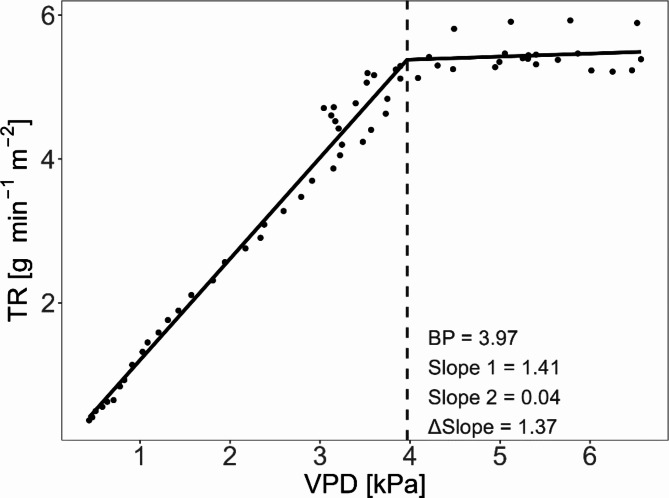
Relationship between transpiration rate and VPD. To demonstrate, the TR response to VPD is visualised for one container on 11 June 2021. TR is plotted against ambient VPD in the DroughtSpotter XXL facility. A linear segmented regression was applied to understand the relationship between transpiration rate and VPD. The vertical dashed line indicates the breakpoint

TR started to increase, along with VPD, at around 6:00. VPD continued to increase until it reached its maximum between 13:00 and 15:00, when TR more or less reached a plateau. TR decreased after 15:00, as the VPD decreased. To characterise this response of TR to the daily cycle of VPD, TR was plotted against VPD and a segmented linear regression model was applied, to identify the VPD breakpoint, Slope 1, Slope 2 and ∆Slope (Fig. 4).

**Fig. 5:**
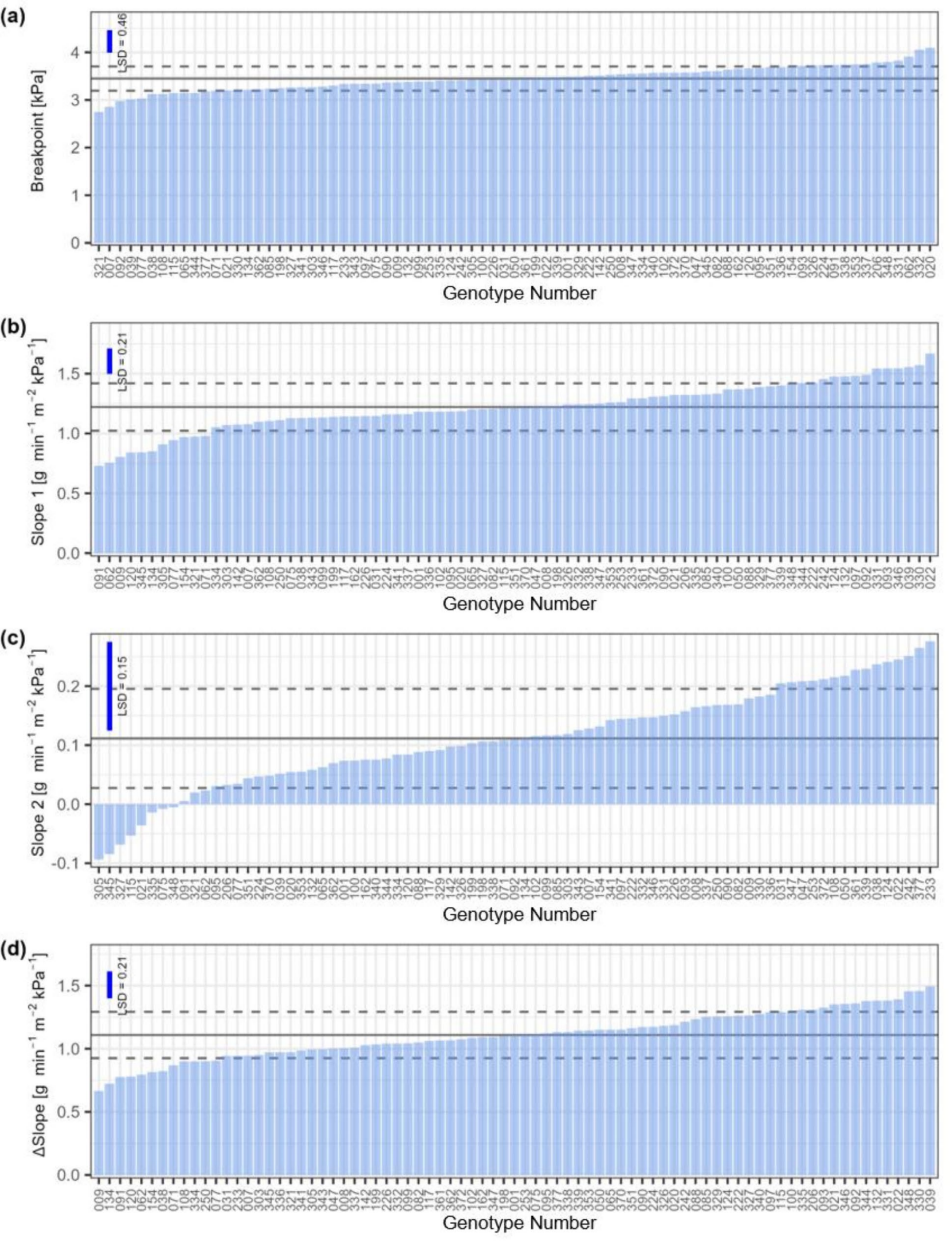
Transpiration response to varying VPD in a set of 79 winter wheat lines tested in the DroughtSpotter XXL. Data show adjusted mean values. The blue vertical line indicates the LSD calculated on a significance level of 10%. The horizontal solid line shows the mean values across all genotypes, the dashed horizontal lines show ± 1SD. (**a**) shows genotypic variation for the breakpoint, (**b**) shows genotypic variation for Slope 1, (**c**) shows genotypic variation for Slope 2 and (**d**) shows genotypic variation for ∆Slope

Adjusted genotype means for the VPD breakpoints ranged from 2.75 kPa to 4.1 kPa. A lower breakpoint might suggest a more conservative use of water. Slope 1 ranged from 0.73 to 1.67 g min^− 1^ m^− 2^ kPa^− 1^. When the VPD exceeds the breakpoint, TR plateaued for most genotypes, resulting in a Slope 2 between − 0.09 and 0.28 g min^− 1^ m^− 2^ kPa^− 1^. Significant genotypic variation was identified for breakpoint, Slope 1, Slope 2 and ∆Slope (*p* = < 0.001), indicated that genotypes differed in their transpiration response to VPD (Fig. 5). Repeatability for these traits was moderate to high (Table 3).

**Table 3:** Variation for the traits derived from segmented regression analysis

Trait	Min	Max	Mean	CoV	Repeatability	Genotype
Breakpoint [kPa]	2.75	4.1	3.45	0.07	0.31	***
Slope 1 [g min^− 1^ m^− 2^ kPa^− 1^]	0.73	1.67	1.22	0.16	0.74	***
Slope 2 [g min^− 1^ m^− 2^ kPa^− 1^]	-0.09	0.28	0.11	0.75	0.27	***
∆Slope [g min^− 1^ m^− 2^ kPa^− 1^]	0.67	1.49	1.11	0.17	0.68	***

Minimum (Min), maximum (Max), mean and the coefficient of variation (CoV) of adjusted mean values for Breakpoint, Slope 1, Slope 2 and ∆Slope. Additionally, repeatability and the statistical significance of the genotypic effect are shown

*, *p* < 0.05; **, *p* < 0.01; ***, *p* < 0.001

There was a significant positive correlation between Slope 1 and Slope 2 (*r* = 0.34, *p* = 0.002, Supplementary Fig. S6). This indicates that genotypes with high transpiration at a low VPD exhibited a higher transpiration at high VPD. Slope 1 and ∆Slope were strongly correlated (*r* = 0.88, *p* < 0.001), indicating that genotypes differ not only in their reaction to contrasting VPD levels and also in their constitutive reaction to VPD (Table 4).

Among the four traits that describe the response of TR to VPD, Slope 1 and Slope 2 were moderately correlated to T (*r* = 0.27, *p* = 0.01 and *r* = 0.27, *p* = 0.02) (Table 4), but no significant correlation to TE was identified. However, a positive moderate correlation between Slope 2 and grain yield was detected (*r* = 0.31, *p* = 0.005), indicating a lower restriction will result in a higher transpiration and therefore higher grain yield due to the strong positive correlation between grain yield and T. Additionally, TGW correlated positively to the breakpoint (*r* = 0.28, *p* = 0.01) and to Slope 1 (*r* = 0.29, *p* = 0.01). The breakpoint was uncorrelated to the other three transpiration response traits, allowing for various combinations between breakpoint and Slope 1, Slope 2 and ∆Slope.

**Table 4:** Pairwise Pearson correlations for transpiration and yield related traits

	BP	S1	S2	∆S	TE	T	GY	BM	TGW	No.Sp	Gr.Sp.
BP											
S1	− .04^ns^										
S2	− .04^ns^	0.34**									
∆S	.02^ns^	0.88***	− .13^ns^								
TE	.01^ns^	− .01^ns^	.13^ns^	− .05^ns^							
T	.22^ns^	0.27*	0.27*	.17^ns^	− .08^ns^						
GY	.13^ns^	.22^ns^	0.31**	.09^ns^	0.58***	0.64***					
BM	.09^ns^	− .07^ns^	.13^ns^	− .12^ns^	.12^ns^	0.73***	0.71***				
TGW	0.28*	0.29*	.07^ns^	0.28*	− .20^ns^	.16^ns^	.03^ns^	.02^ns^			
No.Sp	.04^ns^	.09^ns^	.16^ns^	.02^ns^	0.32**	0.40***	0.51***	0.49***	− 0.26*		
Gr.Sp.	− .02^ns^	− .09^ns^	.11^ns^	− .13^ns^	0.25*	.18^ns^	0.31**	.14^ns^	− 0.31**	− 0.32**	
HI	.11^ns^	0.38***	0.40***	.21^ns^	0.78***	.22^ns^	0.73***	.12^ns^	− .03^ns^	0.31**	0.30**

Pairwise correlations between breakpoint (BP), Slope 1 (S1), Slope 2 (S2), ∆Slope (∆S), transpiration efficiency (TE), cumulative transpiration (T), grain yield (GY), total above ground biomass (BM), thousand grain weight (TGW), number of spikes per container (No.Sp), grains per spike (Gr.Sp.) and harvest index (HI). Significant (*p* < 0.05) correlations are printed in bold

*, *p* < 0.05; **, *p* < 0.01; ***, *p* < 0.001; ns, not significant

#### Clusters of genotypes

To explore how these trait combinations reflect in multispectral traits (Green Leaf Index and Normalized Vegetation Difference Index), K-means clustering based on the traits breakpoint and ∆Slope was applied to identify distinct groups with differing transpiration response to VPD. That way, all genotypes were categorized into four clusters based on their breakpoint and ∆Slope (Supplementary Fig. S7, Supplementary Fig. S8). Cluster “SR” (strong restriction) included 24 genotypes that exhibited a strong transpiration restriction, initiating restriction at a low VPD (low breakpoint) and strongly limited transpiration beyond that breakpoint (high ∆Slope). Cluster “LSR” (late strong restriction) included 15 genotypes that were characterized by a “late” but strong restriction, restricting transpiration only at a high VPD (high breakpoint) but substantially limiting transpiration beyond that breakpoint (high ∆Slope). Cluster “LR” (low restriction) included 26 genotypes that displayed a low transpiration restriction (high breakpoint and low ∆Slope), while Cluster “ELR” (early low restriction) included 14 genotypes which were characterized by an “early” but low restriction (low breakpoint and low ∆Slope) (Table 5).

**Table 5:** Characteristics of the defined clusters

Trait	SR	LSR	LR	ELR
BP [kPa]	3.29 **c** (± 1.5)	3.76 **a** (± 1.5)	3.56 **b** (± 1.3)	3.17 **c** (± 1.8)
Slope 1 [g min^− 1^ m^− 2^ kPa^− 1^]	1.39 **a** (± 0.13)	1.3 **a** (± 0.12)	1.11 **b** (± 0.17)	1.05 **b** (± 0.13)
Slope 2 [g min^− 1^ m^− 2^ kPa^− 1^]	0.11a (± 0.1)	0.08a (± 0.04)	0.12a (± 0.09)	0.13a (± 0.07)
∆Slope [g min^− 1^ m^− 2^ kPa^− 1^]	1.28 **a** (± 0.11)	1.22 **a** (± 0.11)	0.99 **b** (± 0.11)	0.92 **b** (± 0.12)

Mean values for the genotypes in each cluster are shown for the traits breakpoint (BP), Slope 1, Slope 2 and ∆Slope. The letters next to the mean values are significance indicators. Different letters indicate a significant difference between mean values (*p* < 0.05). When the mean values are significantly different, the letters indicate the highest to lowest mean value in alphabetical order. Standard deviations are stated in parentheses next to the mean

**Fig. 6:**
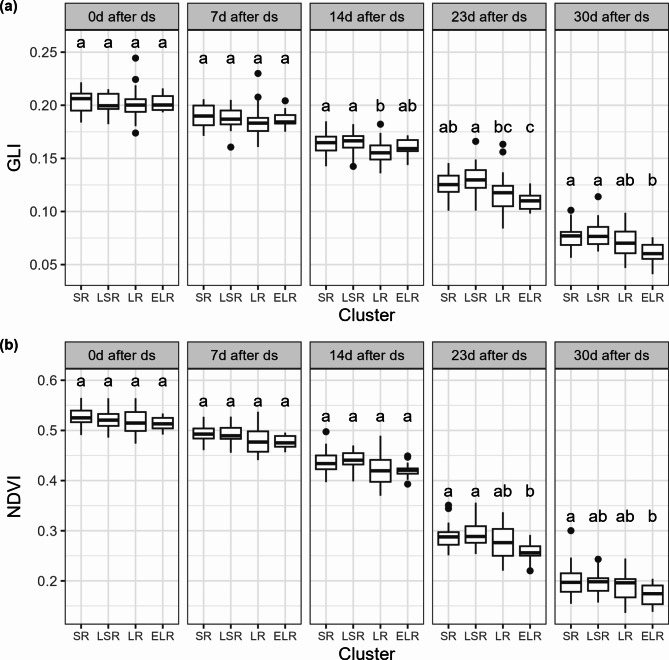
(**a**) Green leaf index (GLI) and (**b**) normalized difference vegetation index (NDVI) on the day of drought stress (ds) application (0d after ds), seven days after drought stress application (7d after ds), 14 days after drought stress application (14d after ds), 23 days after drought stress application (23d after ds) and 30 days after drought stress application (30d after ds). The letters above the boxplots indicate significant (*p* < 0.05) differences between mean values. When the mean values are significantly different, the letters indicate the highest to lowest mean value in alphabetical order

The differing response of transpiration to VPD among the clusters was also reflected by their stay-green characteristics (Fig. 6). LR genotypes showed a significantly lower Green Leaf Index (GLI) than SR and LSR genotypes, at 14 days after drought stress application, whereas SR genotypes maintained a significantly higher GLI than ELR genotypes for up to 23 days after drought stress application (analysis of transpiration response to VPD took place on six days between 17 days after drought stress application and 25 days after drought stress application) (Fig. 6a).

These differences are less pronounced for NDVI. Significantly lower NDVI first appeared 23 days after drought stress application in cluster ELR compared to the SR and LSR genotypes. By 30 days after drought stress application, the genotypes in the SR cluster still exhibited a significantly higher NDVI than the genotypes in cluster ELR, while no significant differences between the other clusters were identified (Fig. 6b).

## Discussion

Evaluating performance of different crop genotypes under drought stress conditions by phenotyping for transpiration and TE is a challenging task because it is difficult to manipulate field drought stress conditions in the required intensity and timing, while it is also difficult to accurately measure transpiration in field environments with the necessary precision and resolution. On the other hand, automated, precise and comprehensive phenotyping of a wide range of traits is possible under controlled environments, but results from controlled-environment experiments are frequently not transferable to a field environment. Large containers can help to “bring the field into the greenhouse” [36, 39, 43], and in contrast to small pots they can allow a planting density and root growth, similar to the conditions in field environments [37, 38]. The DroughtSpotter XXL platform utilised in this study combines large containers with automated, gravimetric “real-time” phenotyping of transpiration, while a dual 3D laser scanner simultaneously enabled monitoring of plant growth and morphological and multispectral traits throughout the crop lifecycle. This combination enabled us to uncover key characteristics and dynamics of transpiration and plant growth and identify genotype-specific differences regarding these traits. This led to the identification of a number of physiological traits related to TE at different stages of development, with high heritability and high expected relevance to field conditions.

### Advantages and restrictions of the phenotyping approach

To the best of our knowledge, this is the first study reporting real-time measurements of transpiration response to changes in VPD in a large panel of wheat genotypes growing under conditions with realistic root growth and plant densities (48 plants per container are equivalent to 300 plants m^− 2^) and therefore conditions with high significance for field environments. Therefore, the experimental setup delivers several advantages in contrast to previous studies conducted either in highly controlled environments like growth chambers, using only small pots that restrict root growth and skew transpiration data, where single plants were tested instead of plant communities, where only a few measurements were taken over a limited time period, where plants were evaluated only in early growth stages or where only few genotypes were tested and compared in detail. The traits describing the transpiration response to VPD and the developmental-stage specific transpiration efficiency showed moderate to very high repeatability (h² = 0.31–0.74 and h² = 0.87–0.91, respectively).

There are also certain limitations of this phenotyping method that need to be critically reflected upon. Even though this system aims at mirroring field environments as closely as possible to enable a high significance for the field it is obvious that the conditions are still very different from the field. Growing conditions do not – as it is usually the case with all comparisons of experiments – represent a perfect copy of the vegetation conditions in the field. Even though larger root volume is provided in this system compared to many other phenotyping platforms that aim at transpiration assessment, the root volume is still smaller than the plants would experience in the field. The major restriction within this system represents the implemented irrigation system. Irrigation to a target container weight means that “water-spending” genotypes receive more water while “water-saving” genotypes are irrigated less [52]. Due to the factual positive correlation of yield and transpiration, water-saving genotypes also showed a lower yield in this experimental set-up. This is a methodological restriction of the watering regime we applied: While transpiration characteristics can be observed easily and precisely, the causal cascade from limited water availability over different genotypic transpiration restriction strategies on grain yield (for example) is not straightforward to compare with the field situation. However, this method allows for a reliable determination of traits that occur in conjunction with this trait. The subtrait “transpiration response to VPD” can be recorded and isolated under conditions of sufficient root depth, a field-like stand density and without other physiological disturbance variables (e.g. hormone response in relation to different degrees of soil moisture removal). A similar phenotyping approach under ambient “outdoor” conditions has already been applied to successfully distinguish the transpiration response to VPD between groups of wild chickpea genotypes and cultivated chickpea genotypes with distinct transpiration efficiency, showing the applicability and effectiveness of such phenotyping approaches for the detection of transpiration reactions to environmental factors [53]. The comparatively high repeatability in our study underlines the high precision of the large-scale system – despite very similar elite wheat genotypes – and the ability of detection of inherited characteristics related to a differential transpiration response. Despite potential limitations, the phenotyping system represents a satisfactory compromise between high-throughput precision phenotyping, with relevance for field conditions, and feasibility.

Phenotyping under realistic and varying conditions is considered decisive for generating meaningful phenotypic data, but every medal has two sides: The dependency on external factors necessitated that the measurement of transpiration response to increasing VPD was contingent upon the occurrence of the appropriate conditions (i.e. high VPD). Consequently, due to the climate conditions while this study was conducted, measurements of transpiration response to VPD could only be performed effectively during grain filling when these conditions were met. This also implies that the results might be difficult to compare to results from other controlled-environment studies, in which plants are usually assessed only in earlier developmental stages. Of course, it remains open whether the results can be attributed to the transpiration response to VPD during earlier stages. Nonetheless, the high repeatability we observed for transpiration response traits underscores the significance of the study as a cornerstone for future research, providing a blueprint for the detailed exploration of other developmental stages.

### Transpiration rate in relation to changes in VPD

The “effective” use of water is agreed to be pivotal for maintaining high performance of crops, especially when water is limited [18, 54], as this results in the conservation of soil water to enable an even water availability throughout the growing season. One key water conservation trait in crops is the restriction of TR in response to elevated VPD. This study evaluated the transpiration response to varying levels of VPD in a large set of winter wheat genotypes and found significant differences among the tested genotypes in the VPD values above which transpiration is restricted as well as in their response in TR to VPD below and above the VPD breakpoint.

Most previous studies of this kind identified two distinct groups of genotypes: genotypes with an unsegmented transpiration response that increased TR in a linear manner along with VPD, and genotypes with a segmented transpiration response which restricted transpiration response at elevated VPD (e.g. 31, 42). In contrast to this, none of the genotypes tested in our study could be clearly associated with a linear unsegmented transpiration response; indeed, all genotypes exposed a breakpoint and therefore a segmented transpiration response to VPD. This might be due to the genotypic proximity of the tested wheat lines, which represent a sample from German commercial breeding programs and are adapted to contemporary climatic conditions in Central Europe.

Genotypic variation for response to VPD has been identified in a large number of crop species, such as soybean [46, 55], maize [42, 56], canola [43], sorghum [57], peanut [58] chickpea [28, 53], pearlmillet [16], and wheat [27, 31, 59]. For bread wheat (*Triticum aestivum* L.), differences in the VPD breakpoint between various wheat lines were detected in a range of 2.4–3.9 kPa and genotypic variation was identified both for transpiration response below the breakpoint as well as above the breakpoint [59]. In a different study with durum wheat, much lower breakpoints of approximately 1 kPa and much less variation between the genotypes was observed [31]. The adaptation of durum wheat to Mediterranean conditions includes improved tolerance to drought and heat stress. The low breakpoint identified in durum wheat [31] aligns with the idea that a strong transpiration restriction in response to VPD enhances performance in water-limited environments. The breakpoints observed for wheat in our study ranged between 2.75 and 4.1 kPa and are only slightly higher than those reported for bread wheat in the above mentioned study [59], but clearly higher than the breakpoints reported for durum wheat [31].

### Association of transpiration response traits with yield traits

Our results showed positive correlations between Breakpoint and TGW as well as between Slope 1 and TGW, and a positive correlation between Slope 2 and grain yield (Table 4). Thus, a lower transpiration restriction, demonstrated by a higher breakpoint and a higher Slope 2, is associated with a higher TGW and grain yield. Furthermore, no correlation was observed between the transpiration restriction traits and TE (Table 4). This appears contradictory, as a higher restriction of TR at elevated VPD would be expected to result in improved TE and higher grain yield compared to genotypes with lower transpiration restriction, under the hypothesis that the restriction of transpiration at unfavourable VPD conditions contributes to water saving. However, due to the irrigation strategy implemented, water-spending genotypes in our experimental setup received a larger quantity of water, resulting in a higher yield compared to water-saving genotypes.

## Conclusions

This study provides a valuable proof of concept for future investigations of transpiration response to VPD using high-resolution, continuous precision phenotyping of water use in crop plants grown under realistic but controlled conditions. High repeatability for VPD response and developmental stage specific transpiration efficiency demonstrates that this kind of facility can provide highly relevant insight into how transpiration dynamics interact across different developmental stages. A future aim is to quantify how these interactions impact yield performance and following that, to identify remote sensing patterns corresponding to stress-induced transpiration dynamics and, therefore, bridge the gap between precision phenotyping studies of this kind (restricted to relatively small population sizes) and field evaluations with larger breeding populations [60]. Knowledge gained in the present study aid identification of the genetic basis of VPD response traits as a basis to facilitate breeding for crops adapted to an altered climate.

## Electronic supplementary material

Below is the link to the electronic supplementary material.

Supplementary Material 1

## Data Availability

The data in support of this study are available from the corresponding author, Anna Moritz, upon request.

## Abbreviations

d: Day
das: Days after sowing
dBM: Digital biomass
ds: Drought stress
dTE: Developmental-stage specific transpiration efficiency
ELR: Early low restriction
FC: Field capacity
GLI: Green leaf index
Gr.Sp: Grains per spike
GY: Grain yield
HI: Harvest index
IQR: Interquartile range
LR: Low restriction
LSD: Least significant difference
LSR: Late strong restriction
Max: Maximum
Min: Minimum
NDVI: Normalised difference vegetation index
No.Sp: Number of spikes
SD: Standard deviation
SR: Strong restriciton
T: Cumulative transpiration
TE: Transpiration efficiency
TGW: Thousand grain weight
TR: Transpiration rate
VPD: Vapour pressure deficit
WU: Water uptake
