# Supplementary material for: Physiological phenotyping of transpiration response to vapour pressure deficit in wheat
Source: BMC Plant Biol. 2024 Oct 30;24:1032. doi: 10.1186/s12870-024-05692-3 (PMC11523787; doi:10.1186/s12870-024-05692-3)
Supplement: Supplementary file 1 — Supplementary Material 1 [file 12870_2024_5692_MOESM1_ESM.docx]

**Supporting Information**

Article title: Physiological phenotyping of transpiration response to vapour pressure deficit in wheat

Authors: Anna Moritz^1^, Andreas Eckert^1^, Stjepan Vukasovic^1^, Rod Snowdon^1^, Andreas Stahl^2^

^1^Department of Plant Breeding, Justus Liebig University Giessen, Giessen, Germany; ^2^Julius Kühn Institute (JKI) – Federal Research Centre for Cultivated Plants, Institute for Resistance Research and Stress Tolerance, Quedlinburg, Germany


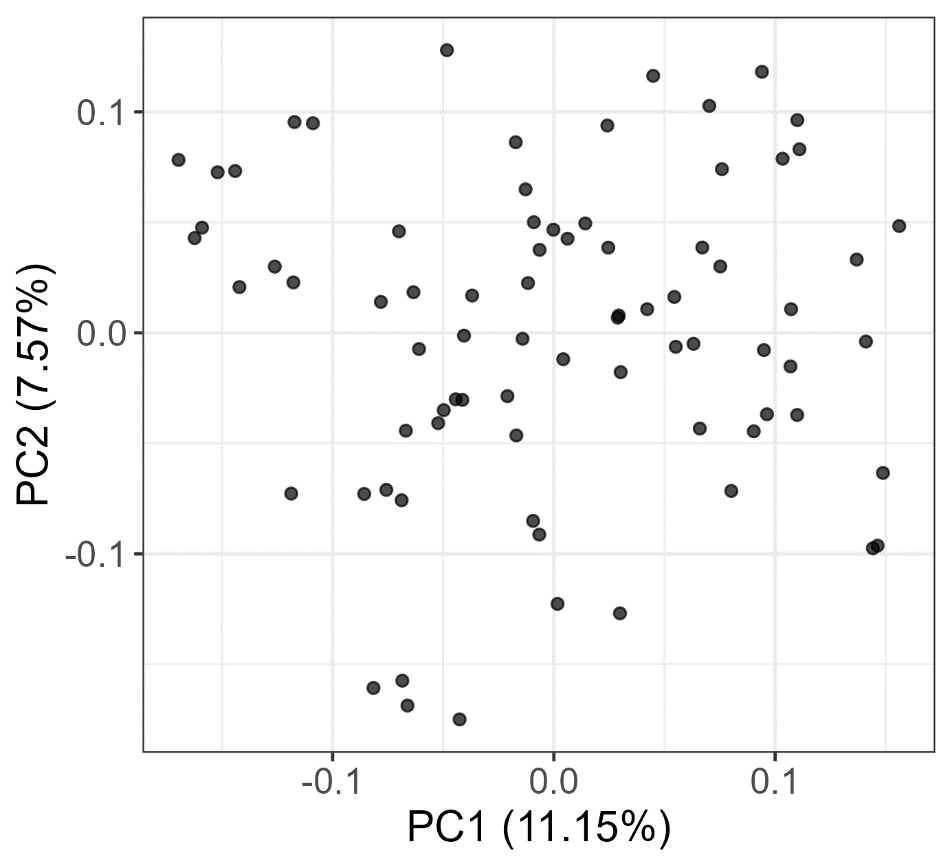


**Fig. S1:** Principal component analysis (PCA) plot illustration the genetic relatedness among the 79 tested wheat genotypes. PCA is based on pairwise Roger’s distances calculated using polymorphic SNP-markers with a minor allele frequency of >5%. The first principal component (PC1) explains 11.15% of the variance within the population, while the second principal component (PC2) explains 7.57% of the variation. No distinct clusters of related genotypes are identifiable.


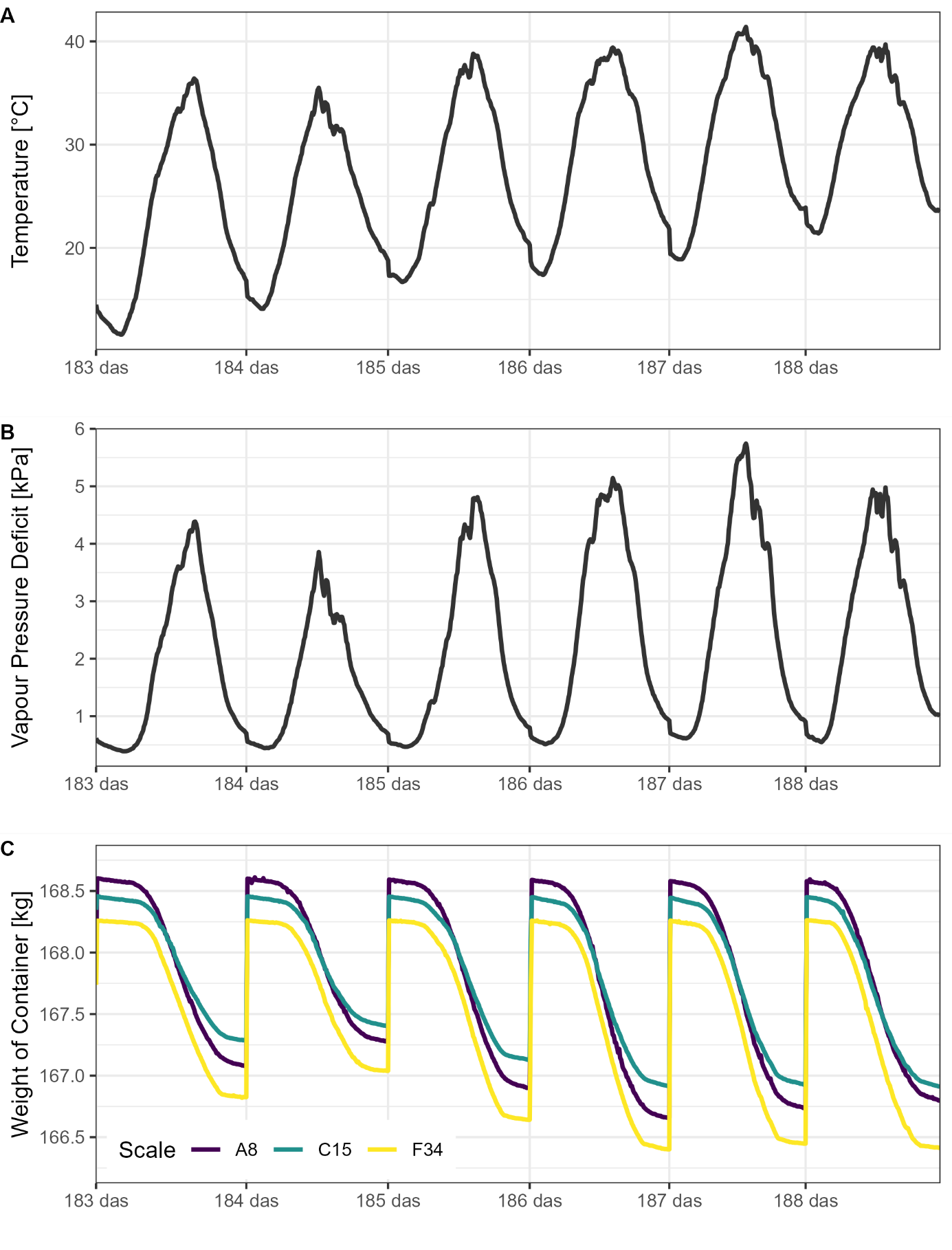


**Fig. S2:** Temperature recordings (A), vapour pressure deficit recordings (B) from the data logger at the central position in the phenotyping facility and the container weight recordings (C) of three arbitrarily selected scales (scale “A8”, scale “C15” and scale “F34”) for six days between 183 days after sowing (das) and 188 das.

**Table S1:** Composition of the nutrition solution applied to each Container

| **Nutrient** | **1^st^ application (80 das) [g Container^-1^]** | **2^nd^ application (102 das) [g Container^-1^]** | **3^rd^ application (156 das) [g Container^-1^]** |
| --- | --- | --- | --- |
| **B** | 0.011 | - | - |
| **Cu** | 0.027 | - | - |
| **K** | 4.512 | 1.786 | 2.680 |
| **Mg** | 0.5 | 0.125 | - |
| **Mn** | 0.053 | - | - |
| **Mo** | 0.005 | - | - |
| **N** | 1.3 | 0.640 | 0.96 |
| **P** | 0.7 | - | - |
| **S** | 0.66 | 0.165 | - |
| **Zn** | 0.107 | - | - |

*
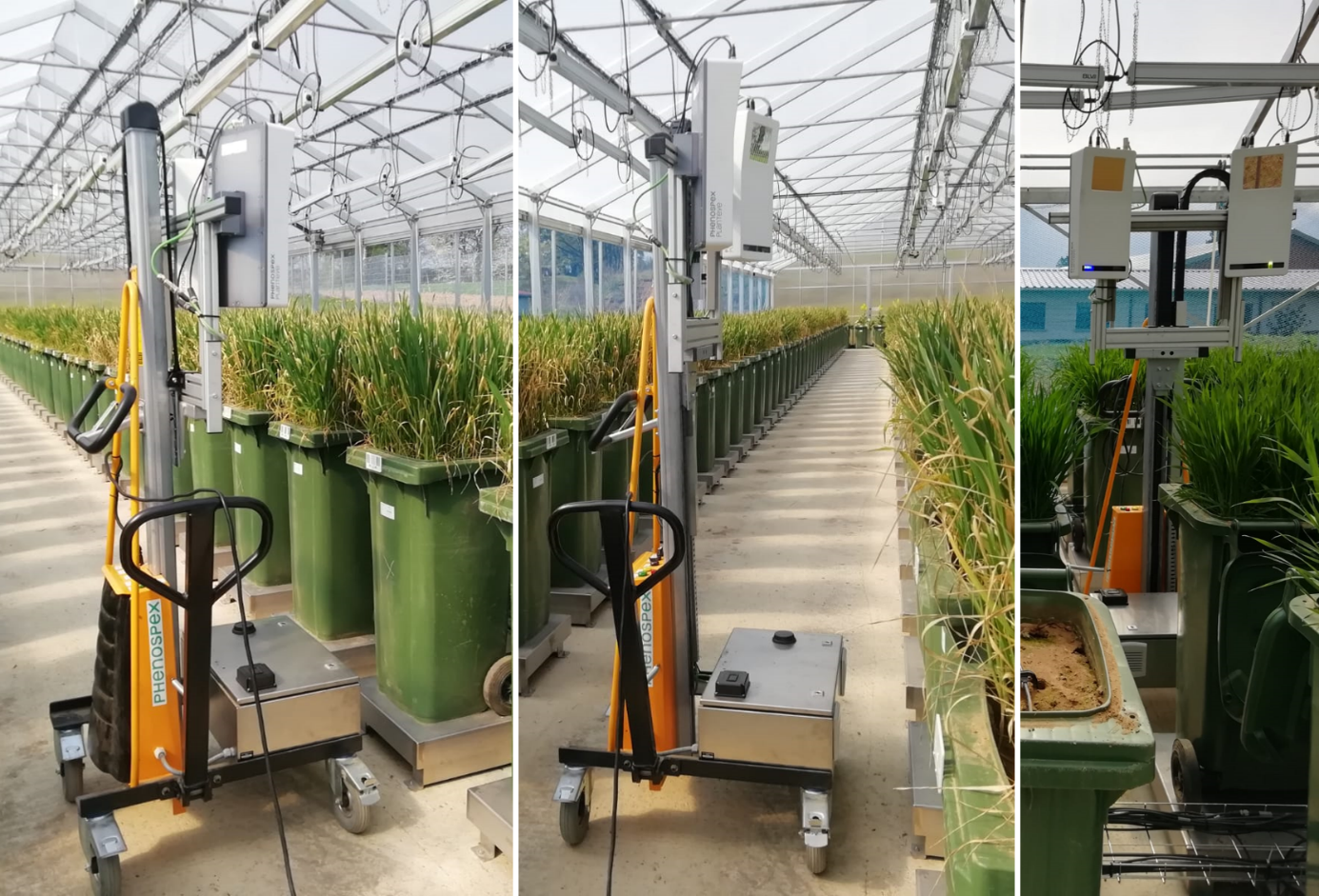
*

**Fig. S3:** Plant Eye F500 3D-Dual Laser Scanner. Depicted from three different angles. The scanning unit is placed in front of a container. After starting a scan, the scanner reads the barcode attached to each container for identification.


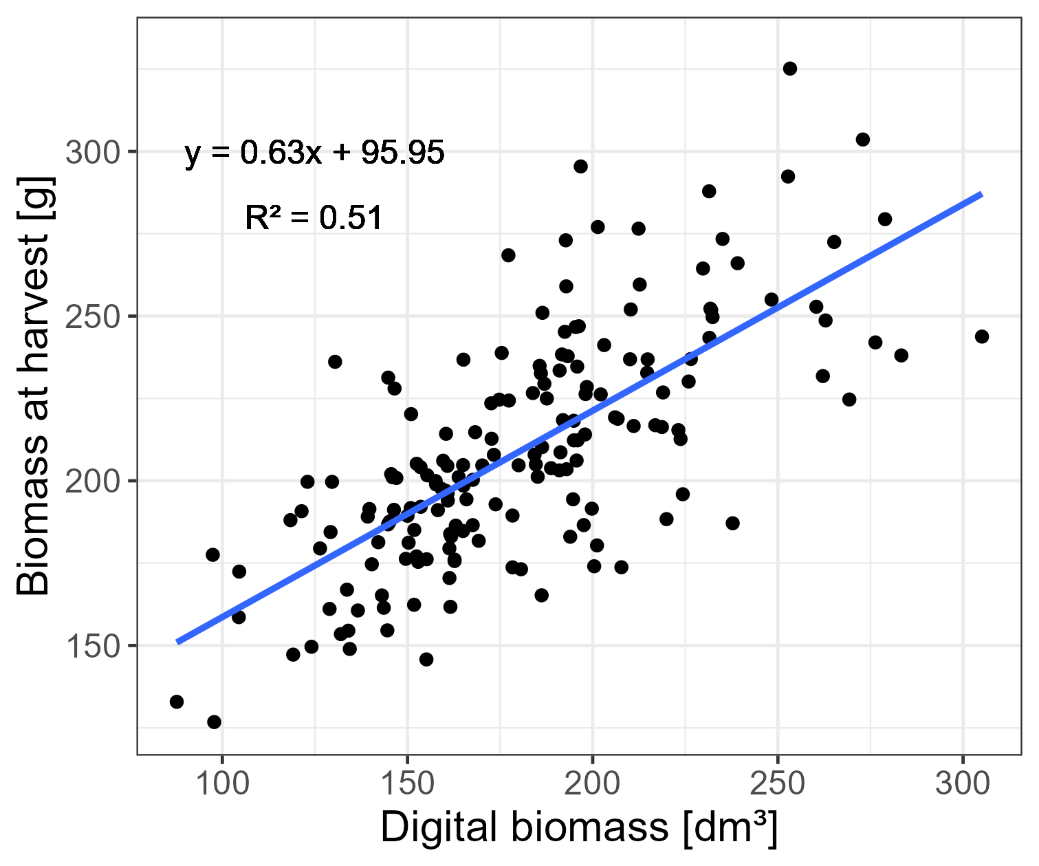


**Fig. S4:** Linear regression of the biomass after harvest with the “digital biomass” estimated with the PlantEye 3D Scanner shortly before harvest of the plants. The adjusted R² (R² = 0.51) and the linear regression equation (y = 0.63x + 95.95) are stated in the figure.


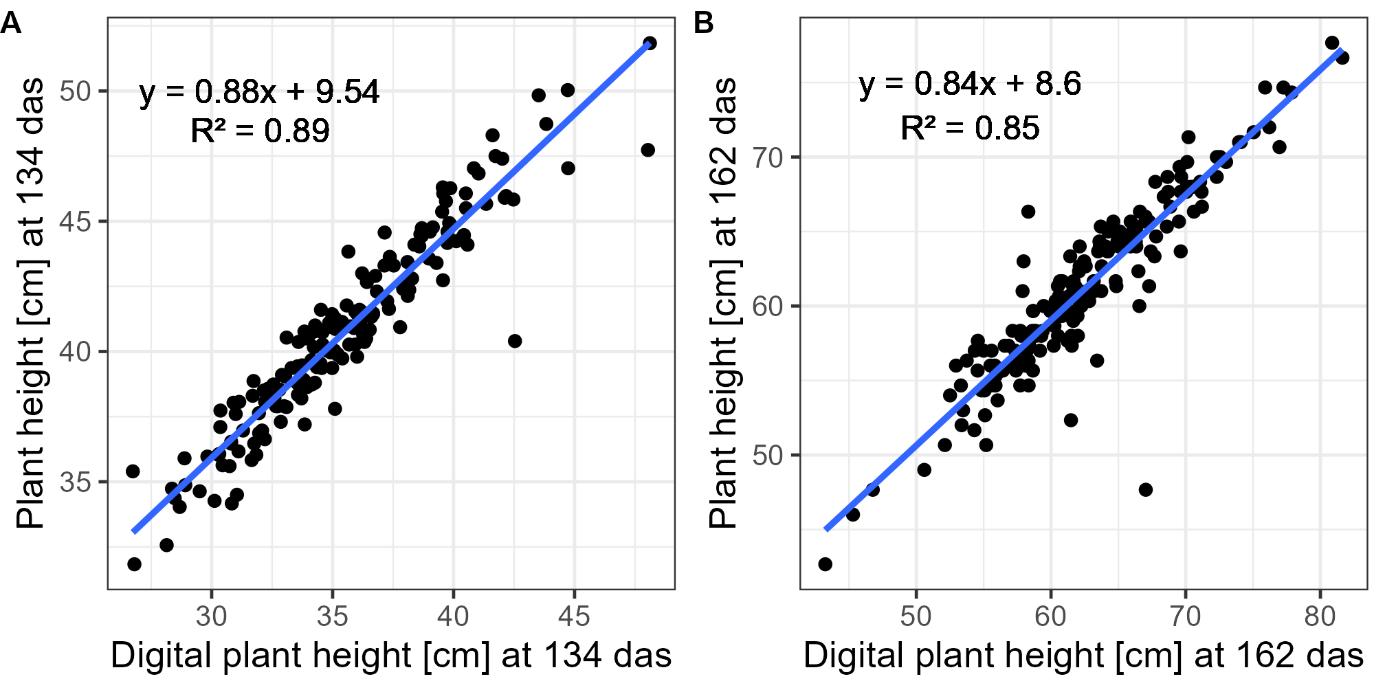


**Fig. S5:** Linear regression of the manually measured plant height using a ruler at (A) 134 das (days after sowing) and (B) 162 das with the “digital plant height” estimated by the PlantEye 3D Scanner at the same date. The adjusted R² (R² = 0.89 for 134 das and R² = 0.85 for 162 das) and the linear regression equations (134 das: y = 0.88x + 9.54 and 162 das: y = 0.84x + 8.6) are stated in the figure.


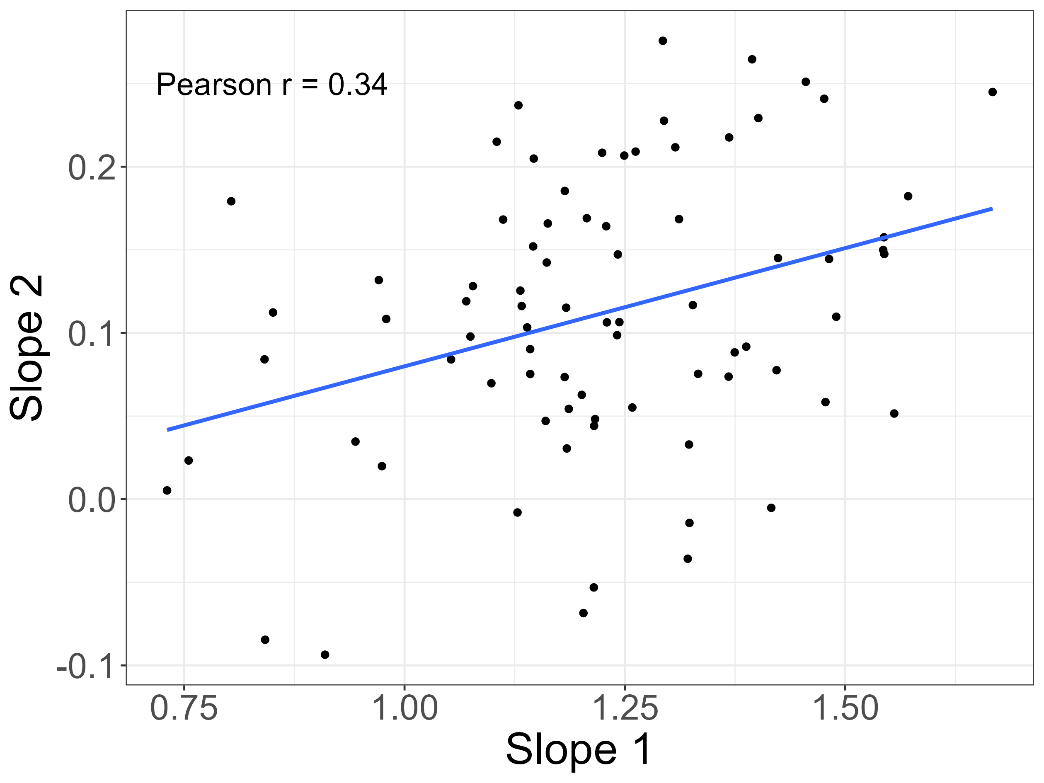


**Fig. S6:** Scatterplot illustrating the correlation between the transpiration response traits slope 1 and slope 2. Each point represents one genotype. The blue line represents the linear regression fit, indicating a positive relationship between the two slopes. The Pearson correlation coefficient is displayed (r = 0.34).


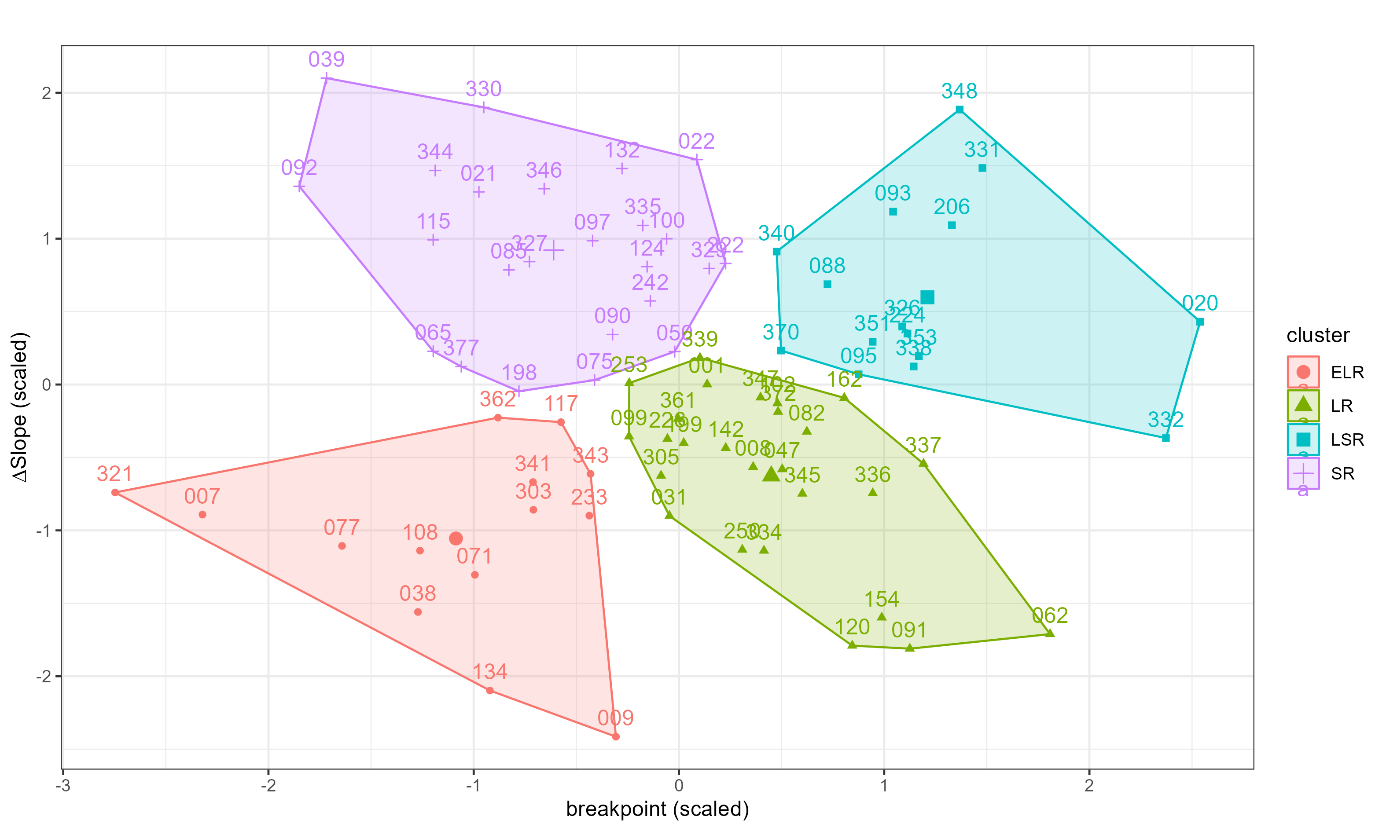
**Fig. S7:** Cluster plot of the genotypes based on their transpiration response to VPD. The scatterplot shows the four cluster groups of genotypes after clustering based on the traits breakpoint and ∆Slope. The clusters are named as follows: “ELR” (early low restriction, orange), “LR” (late restriction, green), LSR (late strong restriction, blue) and “SR” (strong restriction, purple).


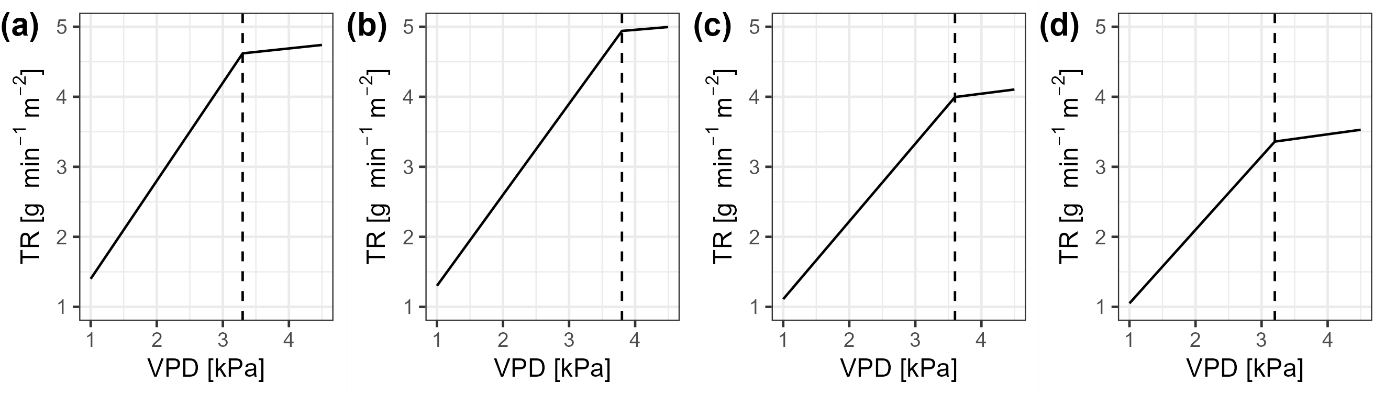


**Fig. S8:** Schematic visualization of the trends of the transpiration rate (TR) in response to vapor pressure deficit (VPD) for the four genotype clusters (a) SR, (b) LSR, (c) LR and (d) ELR. The dashed line corresponds to the breakpoint.
